
JOURNAL INFORMATION
==============================
NLM Title Abbreviation: Northwest Med Surg J
NLM Journal ID: 0020727

The North-Western Medical and Surgical Journal

Publisher: Biomedical Journal Digitization Project

ARTICLE INFORMATION
==============================
PMCID: PMC9973762
PMID: 37320709
Article version: 1
Subjects: Miscellaneous Items

Editorial Changes

Print publication date: 1857 Jul
Volume: 6
Volume Series: New Ser
Issue: 7
First page: 341
Last page: 341
License: This work is free of known copyright restrictions. For more information, please see PMC Back Issue Digitization.
License URL: https://creativecommons.org/publicdomain/mark/1.0/

==============================

*Editorial Changes.*

The editorial management of the New York Journal passes into the hands of Dr. Stephen Smith alone, Drs. Purple and Bulkley withdrawing. The New York Journal is among our ablest cotemporaries, and we wish it abundant success in its future career.

With the June number of the Medical Independent, we find the name of Professor Gunn, of the University of Michigan, associated with Dr. L. G. Robinson, as editors, Dr. Kane having withdrawn.

The last number of the Philadelphia Medical and Surgical Journal completes its fifth volume, and with it Dr. Bryan announces his purpose to suspend the publication, although with some indefiniteness.—*Med. Observer.*

---

**SARGENT & ILSLEY'S**

SOLUTION OF

### CHLORIDE OF ZINC;

*For Purifying Sick Chambers, Sinks, Chamber Vessels, Vaults, Cellars, and for Preserving Corpses, Preventing Moths, Exterminating Bugs, etc.*

To obtain a substance harmless in itself, and free from smell, but possessed of the property of destroying all other smells, particularly such as are offensive or injurious to health, has long occupied the attention of scientific men. It has been found that Chloride of Zinc possesses this power in a high degree, and is also safe, economical and convenient.

It has received the sanction of the highest medical authority, and been very extensively introduced into the hospitals and public institutions of Europe and this country.

Our solution is of uniform strength, containing 35 per cent. of the dry chloride, and for most purposes should be diluted with twenty times its bulk of water. It is the cheapest, most effectual and convenient disinfectant known.

Its application is perfectly safe, both to persons and property; it is also free from the noxious and disagreeable odor of the chloride of lime, and the objections pertaining to the various disinfectants in common use. Full directions accompany each bottle. Prepared by

SARGENT & ILSLEY,

Druggists, 140 Lake street, Chicago.
